# Multi-omics resources for the Australian southern stuttering frog (*Mixophyes australis*) reveal assorted antimicrobial peptides

**DOI:** 10.1038/s41598-024-54522-x

**Published:** 2024-02-18

**Authors:** Simon Tang, Emma Peel, Katherine Belov, Carolyn J. Hogg, Katherine A. Farquharson

**Affiliations:** 1 School of Life and Environmental Sciences, The University of Sydney, Sydney, NSW 2006 Australia; 2 Australian Research Council Centre of Excellence for Innovations in Peptide and Protein Science, The University of Sydney, Sydney, NSW 2006 Australia

**Keywords:** Transcriptomics, Gene expression profiling

## Abstract

The number of genome-level resources for non-model species continues to rapidly expand. However, frog species remain underrepresented, with up to 90% of frog genera having no genomic or transcriptomic data. Here, we assemble the first genomic and transcriptomic resources for the recently described southern stuttering frog (*Mixophyes australis*). The southern stuttering frog is ground-dwelling, inhabiting naturally vegetated riverbanks in south-eastern Australia. Using PacBio HiFi long-read sequencing and Hi-C scaffolding, we generated a high-quality genome assembly, with a scaffold N50 of 369.3 Mb and 95.1% of the genome contained in twelve scaffolds. Using this assembly, we identified the mitochondrial genome, and assembled six tissue-specific transcriptomes. We also bioinformatically characterised novel sequences of two families of antimicrobial peptides (AMPs) in the southern stuttering frog, the cathelicidins and β-defensins. While traditional peptidomic approaches to peptide discovery have typically identified one or two AMPs in a frog species from skin secretions, our bioinformatic approach discovered 12 cathelicidins and two β-defensins that were expressed in a range of tissues. We investigated the novelty of the peptides and found diverse predicted activities. Our bioinformatic approach highlights the benefits of multi-omics resources in peptide discovery and contributes valuable genomic resources in an under-represented taxon.

## Introduction

Since the completion of the Human Genome Project in 2003, there has been a proliferation of high quality genomic and transcriptomic resources for non-model species^1–3^. The ability to generate these ‘multi-omics’ resources in diverse species has clear comparative^4^, conservation^5,6^, and clinical benefits^7^. As of July 2023, over 5800 non-model animal genomes and 4600 non-model animal transcriptomes are available in the largest global genome repository, the National Center for Biotechnology Information (NCBI)^8^. Despite the acceleration of genomic resources, only 46 genera within the Order Anura (10% of all frog genera) have genomic or transcriptomic resources available on NCBI^8,9^. In Australia, despite 92% of frogs being endemic, only 4 of the 229 known endemic frog species currently have ‘omics level resources available^9–13^.

The global disparity in genetic resources across taxa has been driven in part by the diverse structures of tetrapod genomes and associated difficulties with sequencing and assembly^12^. While frogs have relatively stable chromosomal structures compared to other tetrapod groups^14^, they have highly variable genome sizes^10,15,16^; variable proportions of repetitive elements^11,17^; and can have ploidy variation^18^. As a result, the model *Xenopus laevis* genome was one of the only amphibian chromosome-scale assemblies prior to long-read sequencing^19^. More recent advances in long read sequencing technology^20^, alongside chromosome conformation capture techniques^21^, have since facilitated more contiguous assemblies of these challenging genomes^10,22^.

An emerging application of genomic resources is the bioinformatic discovery of novel antimicrobial peptides (AMPs). AMPs are small peptides found across all classes of life that form an important part of the innate immune response^23–25^. The comprehensive characterisation of AMPs is valuable in understanding how different species respond to pathogens in their environment and their pleiotropic immune effects have also been explored as potential therapeutics, with several currently undergoing clinical trials^26,27^. Frogs are known to express AMPs from the exocrine glands of their skin in response to their exposure to diverse, microbial communities in their amphibious, freshwater habitats^28–30^. Over 2500 novel AMPs have been characterised from only 167 frog species on the Database of Anuran Defence Peptides v1.6 (DADP)^31^. To date, frog AMPs have typically been characterised with a peptidomic approach; the iterative purification of AMPs showing antimicrobial activity from frog skin secretions using high-performance liquid chromatography (HPLC)^32^. However, this method may not capture the suite of AMPs expressed in other tissues^33^, nor AMPs with immune functions other than antimicrobial activity^34^. In response, there has been increased interest in ‘mining’ genomic and transcriptomic resources for the genes that encode for AMPs^35^. These methods are primarily homology-driven, using known sequences of large, well-characterised AMP families like cathelicidins and β-defensins to search for homologs^36^. A genomics-driven approach has been applied to discover novel AMPs from a range of non-model species^35,37–39^. However, despite the evolutionary divergence of Australian frog families (Limnodynastidae and Myobatrachidae diverged from South American frogs an estimated 80–100 million years ago)^40,41^, none have yet been investigated for AMPs using a bioinformatic approach.

The southern stuttering frog (*Mixophyes australis*)^42^ is a small, ground-dwelling frog inhabiting naturally vegetated riverbanks in south-eastern Australia, and is part of the Myobatrachidae family of endemic Australian ground frogs^43,44^. The northern (*Mixophyes balbus*) and southern (*Mixophyes australis*) species of stuttering frog were once considered a single species, but were recently split^42^. Although yet to be officially assessed, application of the International Union for the Conservation of Nature (IUCN) threat assessment methods for the southern stuttering frog warrants a listing of ‘Endangered’ for this newly defined species^42^. As a ground-dwelling frog, southern stuttering frogs spend most of their life cycle exposed to large bodies of water and decomposing detritus, which may carry pathogens such as chytrid fungus (*Batrachochytrium dendrobatidis*) and Ranaviruses^42,45^. We hypothesise that the selective pressures of the southern stuttering frog’s environment make it a good candidate for the discovery of novel and diverse AMPs. Preliminary studies on other *Mixophyes* species demonstrated some resistance against chytrid fungus in skin secretions^46,47^. While these secretions have not been sequenced, these studies suggest possible AMP activity in the *Mixophyes* genus.

In this study, we use a combination of PacBio HiFi long-read, short-read, and Hi-C sequencing data to generate the first genomic and transcriptomic resources for the southern stuttering frog. Using these high-quality resources, we use bioinformatics to characterise and analyse the AMP families cathelicidins and β-defensins. By generating the first ‘omics level resources for the *Mixophyes* genus, this study provides a unique opportunity to investigate an otherwise largely understudied, Australian taxon.

## Results

### Multi-omics resources for the southern stuttering frog

**Figure 1:**
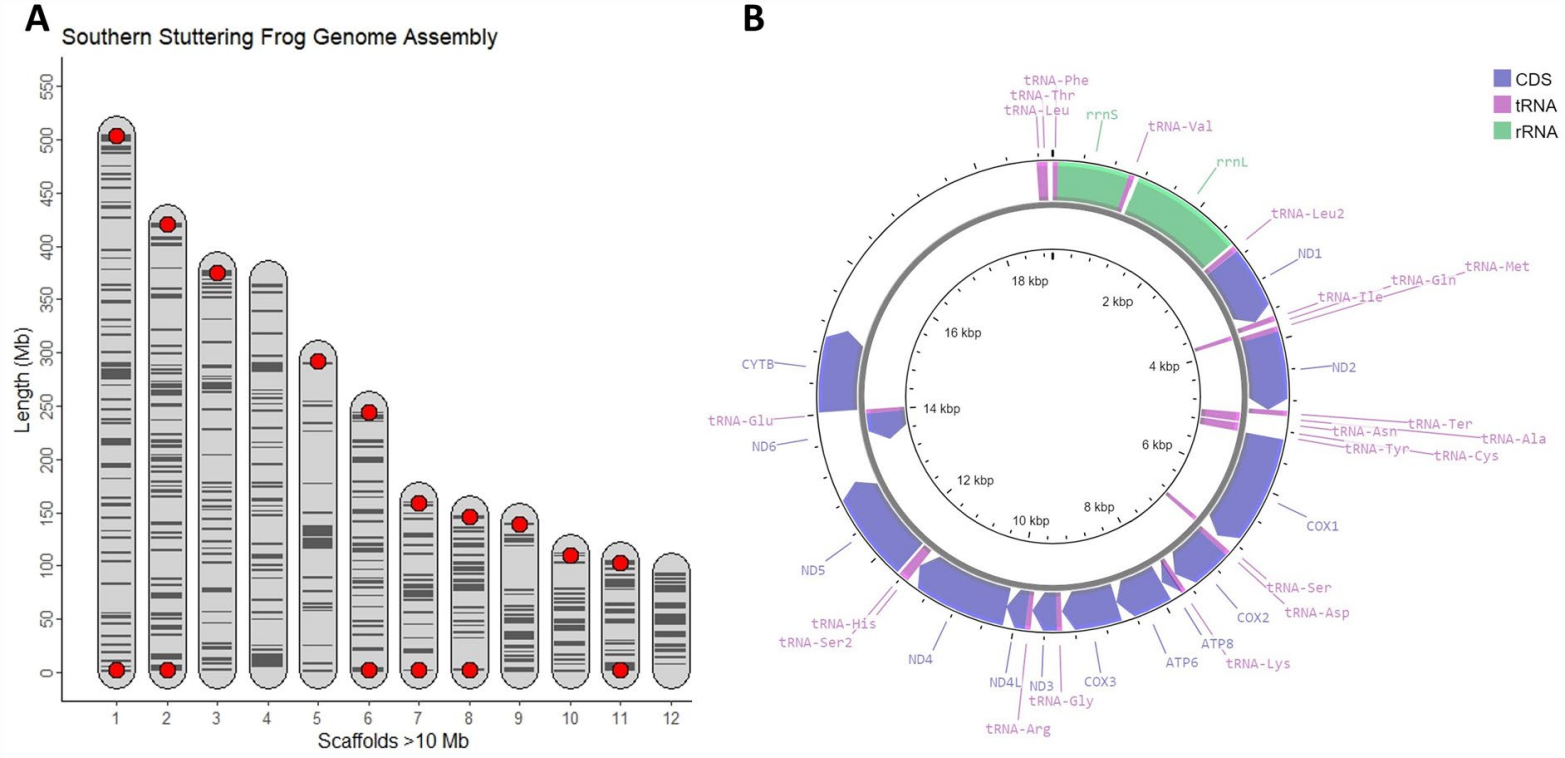
The southern stuttering frog (*Mixophyes australis*) genome assembly. (**A**) The 12 longest southern stuttering frog genome scaffolds, ordered by length. Red dots indicate the presence of telomeric sequences at the end of scaffolds. Horizontal, black lines indicate contig joins informed by the Hi-C sequencing data. (**B**) Circos plot of the southern stuttering frog mitochondrial genome generated using Proksee^48^. Outer ring contains genes on the forward strand, while the inner ring contains genes on the reverse strand.

Using a combination of PacBio HiFi long-read and Hi-C sequencing data, we generated a high-quality genome assembly for the southern stuttering frog (Fig. 1A). The genome is 3.13 Gbp in length, has a read coverage of 26× and a scaffold N50 of 369 Mbp (Table 1). Repeat-masking the genome revealed a high proportion of repetitive elements (53.7%; Supplementary Table S1), within the broad expected range for Anuran genomes (32.0–77.1%;^8^). The GC content of the genome, 40.3%, was also within the range of expected Anuran GC values (26.9–44.5%;^8^). From the assembly, we identified a scaffold representing the mitochondrial genome, which contained 36 genes: 21 tRNAs, 2 rRNAs and 13 protein-coding genes (Fig. 1B).

**Table 1:** Statistics generated from the genome and transcriptomes of the southern stuttering frog (*Mixophyes australis*; this study), the model *Xenopus* species and three Australian frog genome assemblies.

Genome statistics												
Species and assembly number	Size (Gb)		Contig no.		Contig L/N50 (Mb)		Scaffold no.		Scaffold L/N50 (Mb)		Complete BUSCOs (%)	
Southern stuttering frog (*Mixophyes australis*)	3.120		1290		85/11.04		790		4/369.31		91.3	
African clawed frog (*Xenopus laevis*) [GCF_017654675.1]	2.742		716		35/22.45		55		8/155.25		95.9	
Western clawed frog (*Xenopus tropicalis*) [GCF_000004195.4]	1.451		851		32/14.63		167		5/153.96		95.3	
Southern banjo frog (*Limnodynastes. dumerilii*) [GCA_011038615.1]	2.379		739,331		58,116/0.01		520,896		2127/0.29		80.9	
Ornate burrowing frog (*Platyplectrum ornatum*) [GCA_016617825.1]	1.065		238,568		39,409/0.004		148,035		9283/0.03		42.4	
Corroboree frog (*Pseudophryne corroboree*) [GCA_028390025.1]	8.873		5826		353/6.82		3127		5/846.89		93.1	

Transcriptome statistics												
Species	Type			Alignment rate (%)			No. transcripts			Complete BUSCOs (%)		
Southern stuttering frog (*M. australis*)	Brain			86.23			66,201			89.9		
	Gonads (male)			78.62			88,716			93.0		
	Ventral skin			88.32			52,141			89.3		
	Dorsal skin			87.96			55,849			89.8		
	Liver			89.37			46,579			84.0		
	Spleen			83.62			60,664			90.2		
	Global			N/A			192,115			96.1		
African clawed frog (*X. laevis*) [GJQV00000000.1]	Follicle			N/A			333,966			70.8		
Western clawed frog (*X. tropicalis*) [GIVH00000000.1]	Gonads (male and female)			N/A			372,630			95.7		

If available, the NCBI RefSeq or GenBank assembly number is provided. Read alignment rate refers to the percentage of tissue-specific RNA reads that mapped to the genome. BUSCO v5.3.2^49^ scores were calculated in this study for all genomes and transcriptomes using the vertebrata_odb10 lineage. There was no publicly available transcriptome for *L. dumerilii, P. ornatum* and *P. corroboree*, so transcriptome statistics could not be generated for these species. Full BUSCO scores for the genomes, transcriptomes and gene annotations are provided in Table S2.

Over 95% of the genome was assembled in twelve scaffolds (Fig. 1A; Supplementary Fig. S1), which likely represent the chromosomes based on previous karyotypic analysis of other *Mixophyes* species (2n = 24), including *M. hihihorlo*^50^, *M. fasciolatus* and *M. schevilli*^51^. Six scaffolds were flanked at each end by long, telomere-like repeats, while four scaffolds had telomere-like repeats on a single end (Fig. 1A). Additionally, a substantial drop in scaffold length between the 12th and 13th scaffold (94.13 Mbp vs. 4.75 Mbp) was noted, further suggesting 12 chromosome-level scaffolds.

To compare the contiguity and completeness of the genome to other frog genomes, we calculated several genomic statistics, including benchmarking universal single-copy orthologs (BUSCO)^49^ using the vertebrata_odb10 lineage. Genome statistics were calculated for two model frog species and the three publicly available scaffolded Australian frog genomes (Table 1). Our BUSCO analysis revealed that our assembly contained 91.8% of complete BUSCOs, 2.9% were present but fragmented, while 5.3% were missing. The proportion of complete BUSCOs in the southern stuttering frog is slightly lower, but still comparable to the model reference *X. laevis* (95.9%). An unbiased assessment of whole genome completeness with merqury^52^ showed that our genome assembly was highly complete (93.5%) and accurate (Q60+).

Using Illumina short-read RNA sequencing, we generated six reference-guided, tissue-specific transcriptome assemblies (dorsal skin, ventral skin, liver, spleen, brain, and gonads) that were aligned into a global transcriptome. All tissues had high mapping rates to the genome (78.62–89.37%), with both the tissue-specific and global transcriptomes yielding high complete BUSCO scores (84.0–96.1%; Supplementary Table S2). A total of 36,540 genes were annotated using FGENESH++^53^. Of these, 16,355 were annotated using evidence from the coding regions of the global transcriptome, similar to the 16,279 predicted coding regions provided as input. A further 12,892 genes were annotated via homology to non-redundant, metazoan, proteins from NCBI, and 7293 were annotated ab initio. Proteins from these annotated genes yielded a complete BUSCO score of 86.0%, comparable to the genome annotation of *Limnodynastes dumerilii*, and lower than the *Xenopus* spp. annotations (Supplementary Table S2). However, a large proportion of the stuttering frog annotated genes were fragmented (8.5%), similar to *L. dumerilii* (8.0%).

### Characterisation of cathelicidins and β-defensins

We used the genome and transcriptome assemblies of the southern stuttering frog to characterise two families of AMPs: cathelicidins and β-defensins. These families were selected due to their conservation across vertebrate species^54,55^, highly conserved structures and motifs^56–58^ and demonstrated antimicrobial potency^57,59^. We identified 12 cathelicidin (MA-CATH1-12) and two β-defensin (MA-BD1 and MA-BD2) genes in the genome using homology-based search strategies. Full-length transcripts of all cathelicidins and β-defensins were identified in the global transcriptome. All putative AMPs contained the characteristic features of each family, including expected exon number (four for cathelicidins and two for β-defensins), conserved amino acid residues, motifs, and domain structure (Supplementary Figs. S2 and S3). As expected, each AMP family was encoded in clusters within the genome; all cathelicidins were located on scaffold 5 and all β-defensins on scaffold 2 (Supplementary Fig. S4). Cathelicidin and β-defensin genes were named in the order in which they are encoded within the genome (Supplementary Table S3; Fig. S4). Amino acid sequences of all AMPs identified in this study, with predicted signal peptide and mature peptide domains are provided in Supplementary Figs. S2 and S3.

### Properties, evolutionary relationships and expression patterns of cathelicidins and β-defensins

The mature peptide domains of cathelicidins and β-defensins are the bioactive, antimicrobial portion of the peptide. In the southern stuttering frog, the mature peptides of the cathelicidins ranged between 10 and 161 amino acids in length (Supplementary Fig. S2), while the two β-defensins were both 47 amino acids (Supplementary Fig. S3). The mature peptides of AMPs generally have a high positive charge which facilitates electrostatic interaction and attachment to microbial cell membranes^24^. Stuttering frog cathelicidins MA-CATH1, 2 and 3 had a high cationic charge of 8.9, 4.9 and 27.3 respectively at pH 7 (Supplementary Table S4). However, several AMPs identified in this study were weakly cationic (charge 0–3), and MA-CATH8 and MA-CATH2 were anionic. Similarly, while a large proportion (10/14) of the characterised AMPs had > 30% hydrophobic residues, another general trend of AMPs^60^, some peptides like MA-CATH3 had as few as 12.42% hydrophobic residues (Supplementary Table S4). Amphipathicity is often seen in AMPs to permeabilise microbial membranes^60^. Through visual inspection of Kyte and Dolittle hydropathicity plots, almost all the southern stuttering frog AMPs exhibited amphipathicity, with the N-terminus of the peptides being generally more hydrophilic than the C-terminus (Supplementary Fig. S5). MA-CATH3 exhibited some regions of hydrophobicity but was largely hydrophilic (Supplementary Fig. S5).

**Figure 2:**
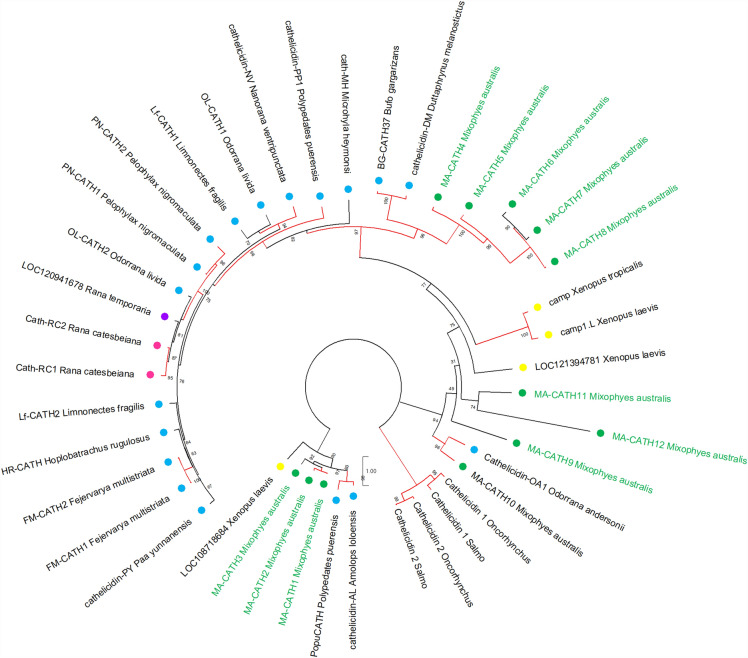
Phylogenetic relationships amongst frog cathelicidins. Tree was generated using the maximum likelihood method, and rooted using four fish cathelicidins as an outgroup. Branches are coloured by ultrafast bootstrap values, with values > 95% in red, and the remaining branches coloured in black. Stuttering frog cathelicidins are coloured green. Cathelicidins are labelled by geographic region; Oceania is green; Asia is blue; North America is pink; Africa is yellow; and Asia and Europe combined is purple. The tree was annotated using MEGA11. For frog cathelicidin sequences used, see Supplementary Table S5.

**Figure 3:**
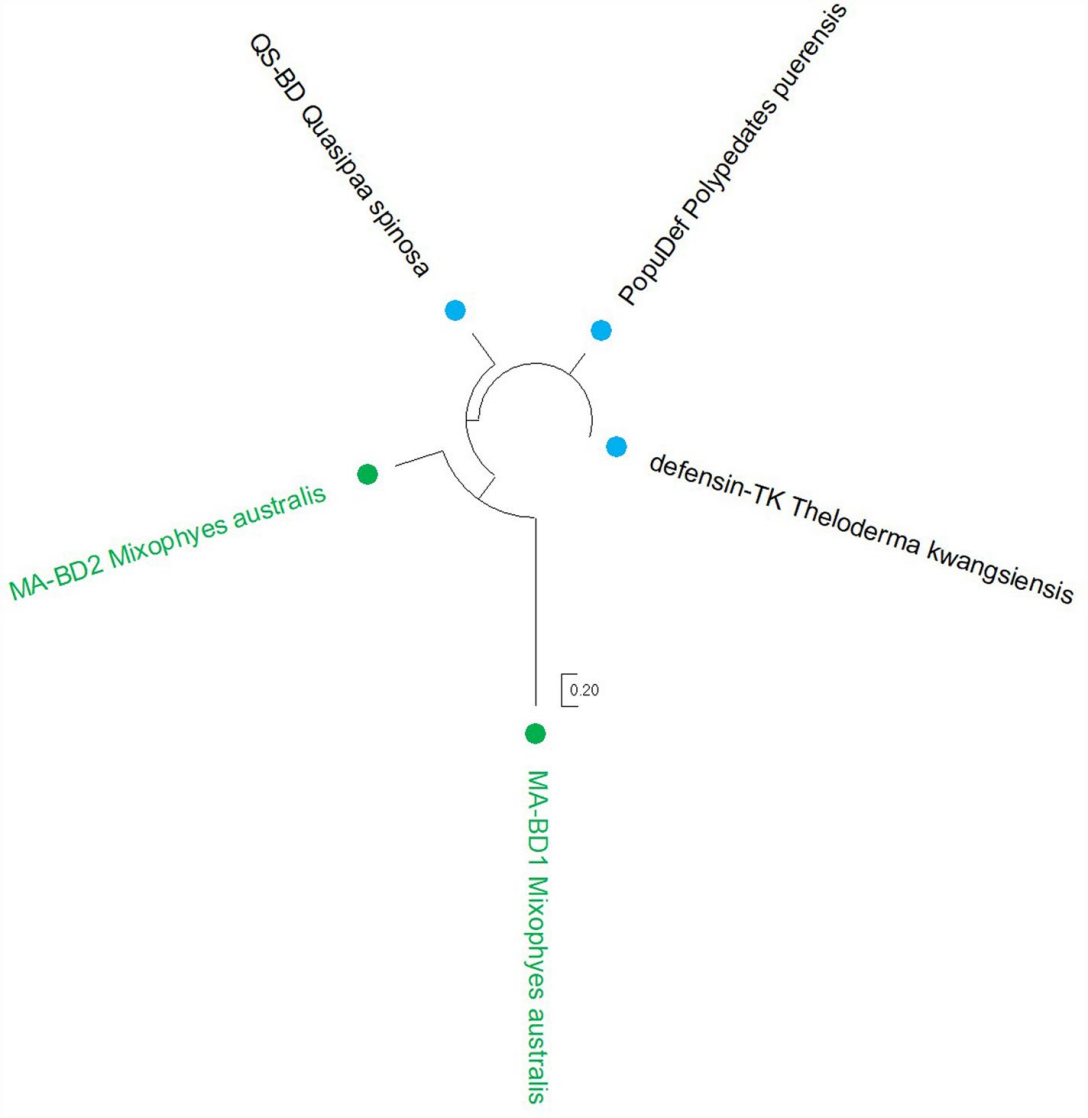
Phylogenetic relationships amongst frog defensins. Tree was generated using the maximum likelihood method and is unrooted. Branches are coloured by ultrafast bootstrap values, with values > 95% in red, and the remaining branches coloured in black. Stuttering frog defensins are coloured green. Defensins are labelled by geographic region; Oceania is green and Asia is blue. The tree was annotated using MEGA11. For frog defensin sequences used, see Supplementary Table S5.

As the first AMPs characterised in the *Mixophyes* genus, we explored their sequence diversity and relationship to other known frog AMPs. All stuttering frog AMPs displayed low percent identity to other known AMPs by BLAST, with MA-BD2 having the highest percent identity of all AMPs in this study to a Chinese spiny frog (*Quasipaa spinosa*) β-defensin (64.15%) (Supplementary Table S4). As expected, maximum likelihood phylogenetic trees generated using all known frog cathelicidins and β-defensins reflected this diversity, as indicated by the long branch lengths (Fig. 2). MA-CATH1-3 and MA-CATH4-8 formed two species-specific clades within the cathelicidin tree, some of which were strongly supported with > 95% ultrafast bootstrap support (Fig. 2). MA-CATH9-12 clustered within clades containing AMPs from multiple Asian and African frog species, albeit with low bootstrap support (Fig. 2). While Fig. 3 suggests that the southern stuttering frog β-defensins are more closely related than the other β-defensins, this relationship was not well supported.

**Figure 4:**
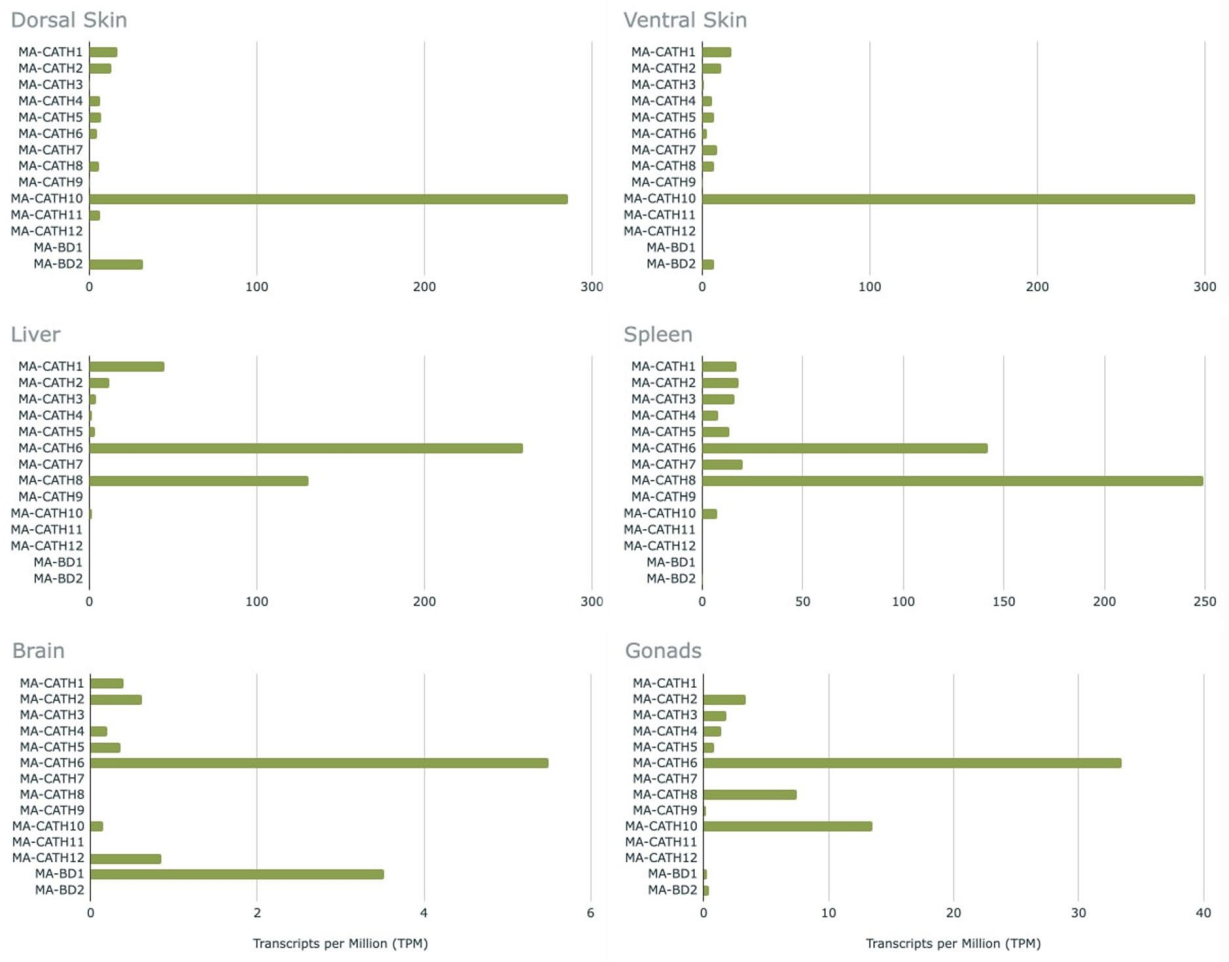
Expression data of the novel cathelicidins (MA-CATH1-12) and β-defensins (MA-BD1-2) from the stuttering frog. Transcripts per Million (TPM) values were derived from the tissue-specific transcriptomes. Note different x-axis scales due to differences in relative expression of AMPs between tissues.

Our comprehensive bioinformatic AMP discovery approach allowed us to explore the expression pattern of AMPs across a range of tissues beyond skin, which is typically the target of peptidomic approaches to AMP discovery. As expected, we observed high AMP gene expression in the dorsal and ventral skin in our specimen. However, we also observed AMP gene expression in other internal organs (Fig. 4). AMP gene expression was found in the liver, spleen, and gonads, with MA-CATH6 and 8 showing the highest expression. MA-CATH10 and MA-BD2 exhibited higher expression in the skin than other AMPs, while some peptides like MA-CATH12 were lowly expressed across all the tissues when compared to other AMPs (Fig. 4).

## Discussion

Here, we used a combination of PacBio HiFi and Hi-C data to generate the first contiguous, annotated, reference genome for the southern stuttering frog. In addition, we assembled the mitochondrial genome and six tissue-specific transcriptomes that were merged into a global transcriptome. The level of resources generated for this single species is comparable with model *Xenopus* species, as few frogs have a publicly available nuclear genome, mitochondrial genome, as well as transcriptome. By comparing genome and transcriptome quality metrics between other Australian and model assemblies, we have also determined that these resources are comparable in contiguity and completeness to the model *Xenopus* assemblies. Additionally, they are the first ‘omics level resources in the *Mixophyes* genus. As the Myobatrachidae family is one of the oldest, most diverse frog families in Australia^40^, these resources add to our understanding of Australian fauna.

To demonstrate the insights that can be gained from these genomic and transcriptomic resources, we bioinformatically characterised 12 cathelicidins and two β-defensins, the first Australian frog AMPs to be discovered using this approach. While other peptidomics-based studies describing cathelicidins in frogs have revealed at most two per species^61,62^, 12 cathelicidins were discovered in this study. Similarly, while one β-defensin has been characterised per species in frogs^57,58^, two were found in this study. In our preliminary gene expression analysis, some of these AMPs, such as MA-CATH1, MA-CATH10 and MA-BD2, were primarily expressed in the dorsal and ventral skin. However, several others had little to no expression in the skin but were primarily expressed in the liver (MA-CATH6), spleen (MA-CATH7-8), and low levels in the brain (MA-BD1). Due to the endangered threat status of the southern stuttering frog, tissue-specific transcriptomes from multiple specimens were not available to determine if these expression patterns are consistent between individuals. However, the number and preliminary expression patterns of the discovered AMPs implies that the peptides that were not expressed in the skin may not have been identified if the more conventional, peptidomics approach of screening skin secretions was used. Our results reveal the benefits of a genomic-based AMP discovery approach and the need for more amphibian genomic resources to characterise such peptides.

It is possible that the evolutionary isolation of Australian amphibians compared to other characterised species has resulted in the diverse suite of cathelicidins and β-defensins observed. Alternatively, the range of cathelicidins and β-defensins characterised here may suggest that the southern stuttering frog is under stronger microbial exposure than other frogs. A previous study on *Diptera* (fly) species has shown that AMP diversity and gene duplication is positively correlated with microbial exposure^25^. This is also likely the case in frogs as they adapt to different global aquatic and terrestrial environments. It is more likely, however, that other frog species and genera may also exhibit a similar number and diversity of cathelicidins and β-defensins which are uncharacterised. The lack of bioinformatic AMP discovery for most frog species makes comparative analysis difficult. Future investigations into frog AMP diversity that incorporate a genomic-based discovery platform will facilitate direct comparisons between species.

Our phylogenetic analysis revealed that some southern stuttering frog cathelicidins formed species-specific sister clades to those containing cathelicidins from frogs of Europe, North America, and Asia (Fig. 2). This may indicate that these particular cathelicidins were the result of gene duplication events that occurred after the southern stuttering frogs diverged from the other frog species within the phylogenetic tree. Indeed, several southern stuttering frog cathelicidins have the same signal and cathelin region, but a different mature peptide. Other southern stuttering frog cathelicidins clustered with cathelicidins from European and African frog species (Fig. 2). This suggests these cathelicidins may be the result of gene duplication events in a more distant common ancestor. However, due in part to the great variability in AMP sequences, many of the relationships identified were not strongly supported, particularly for β-defensins, limiting the validity of insights drawn from these trees. It also remains difficult to ascertain whether the species-specific clusters of southern stuttering frog cathelicidins are truly unique to *M. australis* or the *Mixophyes* genus more broadly, as there are currently no other characterised cathelicidins from *Mixophyes* frogs. The number of known cathelicidins and β-defensins across the Anuran order is limited, with only one cathelicidin characterised in a European frog, and no known cathelicidins or β-defensins from South American frog species. As the Myobatrachidae family shares a distant common ancestor with South American frogs^40^, evolutionary relationships within AMP families across these geographical regions are likely not captured. As more frog cathelicidins and β-defensins are characterised, in particular from Australia and South America, future investigations may better identify the evolutionary patterns of AMP diversity across *Mixophyes* and other frogs.

The characterised and predicted properties of the stuttering frog AMPs suggest that they may play diverse immunological roles. AMPs are generally cationic and amphipathic due to their electrostatic interactions with anionic glycolipids on prokaryotic membranes, which facilitate membrane permeability and cell lysis^63,64^. However, AMPs can also exhibit diverse activities beyond antimicrobial activity and may serve other immune functions. For example, an anionic cathelicidin from a salamander species (TK-CATH) had no tested antimicrobial activity, but instead inhibited pro-inflammatory cytokine gene expression when added to mammalian macrophage cell lines^65^. Two of our AMPs were anionic (MA-CATH8 and MA-BD2; Supplementary Table S4), suggesting that they may have other immune functions. Future investigations will need to validate these in silico findings, such as by synthesising these AMPs and investigating their effects on immune gene expression in a range of cell lines.

While our bioinformatic characterisation of novel AMPs has demonstrated one application of the newly generated genomic and transcriptomic resources, there are numerous other potential applications. For instance, custom DNA metabarcoding markers generated from the mitochondrial genome can now be developed for the southern stuttering frog, contributing to applied conservation outcomes in this threatened species. Metabarcoding has been extensively used to characterise the biodiversity of different environments^66^. Mitochondrial metagenomics (mtMG) has been previously used to distinguish between closely related species of nematodes^67^. As the southern stuttering frog is a recently defined species, closely related to the northern population of stuttering frogs (*M. balbus*)^42^, the mitochondrial genome generated in this study may be a useful monitoring tool, particularly in defining their range and overlap if applicable. Highly contiguous genomes and transcriptomes can also be used to characterise genomic regions that have high repeat content, such as the major histocompatibility complex (MHC)^68^. In frogs, the upregulation of MHC class I and II genes in the Montane brown frog (*Rana ornativentris*) has a functional role in tadpole development^69^. MHC heterozygosity is also a significant predictor of chytrid fungus resistance in the *Lithobates* genus^70,71^. Finally, these resources may be used comparatively with other genomes to investigate conserved and specialised traits across taxa. For example, emerging consortia like the Zoonomia Project have made significant progress in advancing our understanding of mammalian adaptations and evolutionary history^72^. Synteny analyses have previously been conducted in amphibians, but the variation in sequencing quality and gene annotation methods across the limited existing genomes has made deriving insights difficult^73^. Comparative studies in Anurans using high-quality genomic resources from representative genera would advance our understanding of a wealth of unique traits; some frog species can survive in extreme temperatures and environments^74,75^, produce a myriad of toxins^76^, and regenerate lost appendages as tadpoles^77^. The incorporation of the southern stuttering frog genome in future studies into amphibians will facilitate a better representation of evolutionarily unique Australian biodiversity in these investigations. In short, generating high-quality multi-omics resources facilitates a plethora of investigations into the southern stuttering frog and amphibians at large.

## Methods

### Sampling, extractions and sequencing

A wild caught, adult, male stuttering frog was medically euthanised in September 2021 (32° 59′ 52.4″ S 151° 24′ 27.3″ E). Heart and kidney tissue was flash frozen in liquid nitrogen for DNA extraction. Gonads, brain, dorsal skin, ventral skin, liver, and spleen tissue were stored in RNAlater at − 80 °C until RNA extraction. Lethal sampling was conducted under the University of Newcastle Animal Care and Ethics Committee (ACEC Number A-2013-339) and NSW scientific licence (SL190). All methods were performed in accordance with relevant guidelines and regulations; animal research was conducted in compliance with the ARRIVE guidelines.

DNA was extracted from heart and kidney tissue using a Nanobind Tissue Big DNA Kit (Circulomics). Total extracted DNA was verified to be > 20 μg through Qubit fluorometric quantification (ThermoFisher Scientific). DNA was pooled and sequenced at the Australian Genome Research Facility (Brisbane, Queensland, Australia) using a SMRTbell® prep kit 3.0 (PacBio), and circular consensus sequencing (CCS) was performed using three SMRT cells on a PacBio Sequel II system. For Hi-C sequencing, heart and kidney tissue were washed twice for 5 min with 1 × PBS using a rotator wheel at room temperature. Tissues were sequenced at the Biomolecular Resource Facility (Canberra, ACT, Australia), using the Arima Hi-C kit and sequenced as 150-bp paired-end (PE) reads on an Illumina NovaSeq 6000.

RNA was extracted from the six tissues using a Qiagen RNeasy Mini Kit. Concentrations of each sample were confirmed to be ≥ 25 ng/µl using a Nanodrop spectrophotometer (ThermoFisher Scientific) and the RNA integrity number (RIN) measured using the standard Agilent RNA 6000 Nano Kit Protocol and BioAnalyzer (Agilent Technologies). Extractions < 7 RIN were not sequenced. Extracted RNA was prepared at the Ramaciotti Centre for Genomics (Sydney, NSW, Australia), using the Illumina Stranded mRNA Prep Protocol and sequenced as 100-bp PE reads on an Illumina NovaSeq 6000 S1 flowcell.

### De novo genome assembly

Over 15 million raw HiFi reads were generated from three SMRT cells. To prevent low quality reads introducing errors to the assembly, reads with Phred (Q) quality score < 20 were filtered out using bamtools v2.4.1^78^. Reads containing adapter sequence were removed by HiFiAdapterFilt v2.0.0^79^ with default parameters. Further details on the computational requirements and estimated run times for all bioinformatic analyses are provided in Supplementary Table S6. Hifiasm^80^ assembled the remaining reads, alongside paired Hi-C reads, into contigs. To merge the contigs into a scaffolded assembly, we first mapped the Hi-C reads to the unscaffolded genome following the Arima Hi-C mapping pipeline (A160156 v02; https://github.com/ArimaGenomics/mapping_pipeline). YaHS^81^ was used to merge contigs containing complementary pairs of reads, and the contact map visualised with Juicebox^82^. One misassembly in the first scaffold was manually corrected. We used MitoHiFi v3.2 to identify and annotate the mitochondrial genome using a closely-related mitogenome as input (*Lechriodus melanopyga*; NC_019999.1)^83,84^.

Genome statistics (e.g., N50 and L50 values), were calculated with bbmap v38.86 (https://sourceforge.net/projects/bbmap/). We identified regions matching canonical telomere hexamer repeats (TTAGGG/CCCTAA) using FindTelomeres (https://github.com/JanaSperschneider/FindTelomeres). Benchmarking Universal Single-Copy Orthologs (BUSCO) v5.3.2^49^ analysis was performed on Galaxy Australia^85^, using the ‘genome’ mode, applying the ‘augustus’ gene-finding setting, and with the vertebrata_odb10 lineage. To compare the gene completeness of the stuttering frog with other genomes, BUSCO analysis was also performed with the same settings on two model frog genomes, the African clawed frog (*Xenopus laevis*; GCA_017654675.1) and the western clawed frog (*Xenopus tropicalis*; GCA_000004195.4), as well as the three publicly available scaffolded Australian frog genomes, the southern banjo frog (*Limnodynastes dumerilii*; GCA_011038615.1)*,* ornate burrowing frog (*Platyplectrum ornatum*; GCA_016617825.1), and corroboree frog (*Pseudophryne corroboree*; GCA_028390025.1). An alternative assessment of genome completeness, inclusive of non-coding and repetitive regions, was performed for the stuttering frog and *P. ornatum* genomes using Merqury v1.3^52^. RepeatModeler v2.0.1^86^ was used to generate a de novo database of the repetitive regions in the stuttering frog genome. We characterised repeats and masked the genome with RepeatMasker v4.0.6^87^. The repeat-masked genome was indexed with hisat2 v2.1.0^88^.

## Reference-aligned global transcriptome assembly

Over 900 million PE reads were generated across the six tissue transcriptomes. Low quality sequence calls, adapter and primer sequences were trimmed using Trimmomatic v0.39^89^ with ILLUMINACLIP:TruSeq3-PE.fa:2:30:10, SLIDINGWINDOW:4:5, LEADING:5, TRAILING:5, and MINLEN:25 settings. The reads from each tissue sample were aligned to the genome with hisat2 v2.1.0^88^. StringTie v2.1.6^90^ was used to merge aligned reads into tissue-specific transcriptomes. We used Transcriptome Annotation by Modular Algorithms (TAMA) v1.0^91^ to combine the tissue-specific transcriptomes into a global transcriptome with adjustments to minimise duplicate transcripts. Briefly, the ‘-d merge_dup’ flag was applied to merge identical transcripts, and the ‘-z 500’ flag was applied to facilitate transcripts with variable 3’ ends (differences of up to 500 bp) to be merged. Transcripts with weak evidence were removed, including transcripts that were found in only one tissue and were lowly expressed (fragments per kilobase of transcript per million fragments mapped [FPKM] < 0.1). CPC2 v2019-11-19^92^ was used to predict coding regions; non-coding regions were removed. Open reading frames were predicted by TransDecoder v2.0.1^93^. BUSCO v5.3.2^49^ analysis was performed using ‘transcriptome’ mode for both tissue-specific transcriptomes and the global transcriptome, as well as the *X. tropicalis* transcriptome for comparison.

### Genome annotation

The genome was annotated with FGENESH++ v7.2.2^53^ informed by the global transcriptome. Non-mammalian settings were applied throughout the pipeline, and parameters were optimised for Anuran gene discovery by providing the *Xenopus* gene-finding matrix (Softberry). BUSCO v5.3.2^49^ analysis was performed on the annotation using ‘protein’ mode, as well as for the *X. laevis, X. tropicalis* and *L. dumerilii* annotations. The number of genes, exons and introns from the stuttering frog annotated assembly was calculated using the ‘genestats’ script (https://github.com/darencard/GenomeAnnotation/blob/master/genestats).

### AMP characterisation

To ensure a comprehensive search of genomic regions that could encode cathelicidins or β-defensins, the annotated genome and global transcriptome were queried using Basic Local Alignment Search Tool (BLAST) v2.2.30+^94,95^ and HMMER v3.3^96^. A dual program approach has previously been applied in other bioinformatic searches for cathelicidins and β-defensins^97–99^. Further explanation of the approaches used is provided in the Supplementary Extended Methods.

### AMP phylogeny

An unrooted phylogenetic tree was generated for the cathelicidin and β-defensin gene families. We first aligned amino acid sequences for each gene family through ClustalW alignments. Both alignments included the full prepropeptide sequences from the stuttering frog and other available frogs (Supplementary Table S5). The cathelicidin alignment also included four fish cathelicidins as an outgroup (Supplementary Table S5). The best-fitting substitution models for each alignment were selected using the ModelFinder option in IQ-TREE v2.2.0^100^ according to the Bayesian information criterion^101^. For the cathelicidin alignment, the Jones Taylor Thornton (JTT) substitution model^102^ was optimal, incorporating Invariant Sites (+ I) and four components of gamma rate heterogeneity (+ G4). For the β-defensin alignment, the Dayhoff model was optimal^103^. Maximum likelihood analysis was conducted in IQ-TREE v2.2.0^100^ with node support estimated by ultrafast bootstrap approximation with 1000 replicates^104^. The results were visualised and annotated using MEGA11^105^.

### Characterisation and prediction of AMP structure, properties and expression

Cathelicidins are made up of a signal peptide, a conserved cathelin pro-region, as well as the mature, bioactive peptide^56,106^. To predict the signal peptide from the full prepropeptide sequence, the SignalP v6.0 webserver was used^107^ with the ‘Eukarya’ organism setting and the ‘Slow’ model mode. While the enzyme used to cleave the mature peptide from the cathelin pro-region is not known for amphibians, there is experimental evidence to support proprotein convertases or trypsin-like proteases that cleave at dibasic residues like lysine (K) and arginine (R)^61,108,109^. Therefore, a two-tiered approach predicted the mature peptide of the novel cathelicidins. Proprotein convertase cleavage sites were first predicted from the last exon of the cathelicidins using ProP v1.0^110^. If no cleavage was identified, trypsin cleavage sites were predicted using ExPASy’s peptide cutter tool^111^. For β-defensins, which generally consist of a signal peptide and a mature, bioactive peptide^112–114^, the signal peptide was predicted using SignalP, and the remaining peptide was annotated as the mature peptide.

The molecular weight and charge at pH 7 for each putative stuttering frog cathelicidin and β-defensin was calculated using Protein Calculator v3.4 (https://protcalc.sourceforge.net/). The percentage of hydrophobic residues in the AMPs was calculated on Peptide v2.0 (https://www.peptide2.com/) using the ‘Peptide Hydrophobicity/Hydrophilicity Analysis’ tool. The amphipathicity of the AMPs was determined by generating Kyte and Doolittle hydropathicity plots on ExPASy using Protscale^111^, with a window size of 5. These plots were inspected for the presence of ‘peaks’ and ‘troughs’, which indicate sections in the AMP of high and low hydropathicity^115^. For the expression analysis, the Transcripts Per Million (TPM) values for each AMP was generated from the tissue-specific transcriptomes using StringTie v2.1.6^90^.

### Supplementary Information

Supplementary Information.

## Data Availability

The genome assembly, mitochondrial genome, and raw transcriptome sequencing data generated in this study have been submitted to the NCBI BioProject database (https://www.ncbi.nlm.nih.gov/bioproject/) under accession number PRJNA991157. Raw genome sequencing data is publicly available through the Bioplatforms Australia Threatened Species Initiative (https://data.bioplatforms.com/organization/threatened-species). The global transcriptome and annotation generated in this study are available on Amazon Web Services Australasian Genomes Open Data Store (https://awgg-lab.github.io/australasiangenomes/genomes.html).

## References

[1] Lewin HA (2018). Earth BioGenome Project: Sequencing life for the future of life. Proc. Natl. Acad. Sci..

[2] Lewin HA (2022). The Earth BioGenome Project 2020: Starting the clock. Proc. Natl. Acad. Sci. U S A.

[3] Hotaling S, Kelley J, Frandsen P (2021). Toward a genome sequence for every animal: Where are we now?. Proc. Natl. Acad. Sci..

[4] Horgan R, Kenny L (2011). ‘Omic’ technologies: Genomics, transcriptomics, proteomics and metabolomics. Obstet. Gynaecol..

[5] Formenti G (2022). The era of reference genomes in conservation genomics. Trends Ecol. Evol..

[6] Paez S (2022). Reference genomes for conservation. Science.

[7] Wong AK (2021). Decoding disease: From genomes to networks to phenotypes. Nat. Rev. Genet..

[8] Sayers EW (2022). Database resources of the national center for biotechnology information. Nucleic Acids Res..

[9] AmphibiaWeb (2022). Amphibian Species by the Numbers.

[10] Lamichhaney S (2021). A bird-like genome from a frog: Mechanisms of genome size reduction in the ornate burrowing frog, *Platyplectrum ornatum*. Proc. Natl. Acad. Sci. USA.

[11] Li Q (2020). A draft genome assembly of the eastern banjo frog *Limnodynastes dumerilii dumerilii* (Anura: Limnodynastidae). Gigabyte.

[12] Rhie A (2021). Towards complete and error-free genome assemblies of all vertebrate species. Nature.

[13] Farquharson K (2023). The genome sequence of the critically endangered Kroombit tinkerfrog (*Taudactylus pleione*) [version 1; peer review: 2 approved]. F1000Research.

[14] Bredeson JV (2024). Conserved chromatin and repetitive patterns reveal slow genome evolution in frogs. Nat. Commun..

[15] Liedtke HC (2018). Macroevolutionary shift in the size of amphibian genomes and the role of life history and climate. Nat. Ecol. Evol..

[16] Sun Y-B, Zhang Y, Wang K (2020). Perspectives on studying molecular adaptations of amphibians in the genomic era. Zool. Res..

[17] Seidl F (2019). Genome of *Spea multiplicata*, a rapidly developing, phenotypically plastic, and desert-adapted spadefoot toad. G3 Genes Genomes Genetics.

[18] Novikova PY (2020). Polyploidy breaks speciation barriers in Australian burrowing frogs *Neobatrachus*. PLoS Genet..

[19] Session AM (2016). Genome evolution in the allotetraploid frog *Xenopus laevis*. Nature.

[20] Pollard MO (2018). Long reads: Their purpose and place. Hum. Mol. Genet..

[21] Lieberman-Aiden E (2009). Comprehensive mapping of long-range interactions reveals folding principles of the human genome. Science.

[22] Streicher JW (2021). The genome sequence of the common frog, *Rana temporaria* Linnaeus 1758. Wellcome Open Res..

[23] Wang G, Li X, Wang Z (2016). APD3: The antimicrobial peptide database as a tool for research and education. Nucleic Acids Res..

[24] Huan YC (2020). Antimicrobial peptides: Classification, design, application and research progress in multiple fields. Front. Microbiol..

[25] Hanson MA, Lemaitre B, Unckless RL (2019). Dynamic evolution of antimicrobial peptides underscores trade-offs between immunity and ecological fitness. Front. Immunol..

[26] Mercer DK (2020). NP213 (Novexatin®): A unique therapy candidate for onychomycosis with a differentiated safety and efficacy profile. Med. Mycol..

[27] Ridyard KE, Overhage J (2021). The potential of human peptide LL-37 as an antimicrobial and anti-biofilm agent. Antibiotics.

[28] Wang Q (2020). Diversity of antimicrobial peptides in three partially sympatric frog species in Northeast Asia and implications for evolution. Genes (Basel).

[29] Varga JFA, Bui-Marinos MP, Katzenback BA (2018). Frog skin innate immune defences: Sensing and surviving pathogens. Front. Immunol..

[30] Ladram A, Nicolas P (2016). Antimicrobial peptides from frog skin: Biodiversity and therapeutic promises. Front. Biosci. Landmark.

[31] Novković M (2012). DADP: The database of anuran defense peptides. Bioinformatics.

[32] Yang Y (2022). A non-bactericidal cathelicidin provides prophylactic efficacy against bacterial infection by driving phagocyte influx. eLife.

[33] Hao X (2012). Amphibian cathelicidin fills the evolutionary gap of cathelicidin in vertebrate. Amino Acids.

[34] He X (2019). A frog-derived immunomodulatory peptide promotes cutaneous wound healing by regulating cellular response. Front. Immunol..

[35] Peel E (2016). Cathelicidins in the Tasmanian devil (*Sarcophilus harrisii*). Sci. Rep..

[36] Dalla Valle L (2012). Bioinformatic and molecular characterization of beta-defensins-like peptides isolated from the green lizard *Anolis carolinensis*. Dev. Comp. Immunol..

[37] Wang M (2021). Identification and characterization of antimicrobial peptides from butterflies: An integrated bioinformatics and experimental study. Front. Microbiol..

[38] Pérez de la Lastra JM (2021). Bioinformatic analysis of genome-predicted bat cathelicidins. Molecules.

[39] Yoo WG (2015). Genome-wide identification of antimicrobial peptides in the liver fluke, *Clonorchis sinensis*. Bioinformation.

[40] Brennan IG (2023). Populating a continent: Phylogenomics reveal the timing of Australian frog diversification. Syst. Biol..

[41] Irisarri I (2012). The origin of modern frogs (*Neobatrachia*) was accompanied by acceleration in mitochondrial and nuclear substitution rates. BMC Genomics.

[42] Mahony M (2023). A new species of barred frog, Mixophyes (Anura: Myobatrachidae) from south-eastern Australia identified by molecular genetic analyses. Zootaxa.

[43] Barker J, Grigg G, Tyler MJ (1995). A Field Guide to Australian frogs.

[44] Cogger HG (2000). Reptiles and Amphibians of Australia.

[45] Murray BR, Hose GC (2005). Life-history and ecological correlates of decline and extinction in the endemic Australian frog fauna. Austral Ecol..

[46] Woodhams DC (2007). Resistance to chytridiomycosis varies among amphibian species and is correlated with skin peptide defenses. Anim. Conserv..

[47] Hollanders M (2023). Recovered frog populations coexist with endemic *Batrachochytrium dendrobatidis* despite load-dependent mortality. Ecol. Appl..

[48] Grant JR (2023). Proksee: In-depth characterization and visualization of bacterial genomes. Nucleic Acids Res..

[49] Simão FA (2015). BUSCO: Assessing genome assembly and annotation completeness with single-copy orthologs. Bioinformatics.

[50] Donnellan SC, Mahony MJ, Davies M (1990). A new species of mixophyes (Anura: Leptodactylidae) and first record of the genus in New Guinea. Herpetologica.

[51] Schmid M (2002). Chromosome banding in Amphibia. XXV. Karyotype evolution and heterochromatin characterization in Australian Mixophyes (Anura, Myobatrachidae). Cytogenet. Genome Res..

[52] Rhie A (2020). Merqury: Reference-free quality, completeness, and phasing assessment for genome assemblies. Genome Biol..

[53] Solovyev V (2006). Automatic annotation of eukaryotic genes, pseudogenes and promoters. Genome Biol..

[54] Kosciuczuk EM (2012). Cathelicidins: Family of antimicrobial peptides. A review. Mol. Biol. Rep..

[55] Tu J (2015). Molecular evolutionary analysis of β-defensin peptides in vertebrates. Evol. Bioinform. Online.

[56] Chen J (2021). Molecular characterization of cathelicidin in tiger frog (*Hoplobatrachus rugulosus*): Antimicrobial activity and immunomodulatory activity. Comp. Biochem. Physiol. Part C Toxicol. Pharmacol..

[57] Shen W (2016). A novel defensin-like antimicrobial peptide from the skin secretions of the tree frog, *Theloderma kwangsiensis*. Gene.

[58] Yu SS (2022). Antimicrobial and immunomodulatory activity of beta-defensin from the Chinese spiny frog (*Quasipaa spinosa*). Dev. Comp. Immunol..

[59] Wei L (2015). The first anionic defensin from amphibians. Amino Acids.

[60] Chen CH, Lu TK (2020). Development and challenges of antimicrobial peptides for therapeutic applications. Antibiotics.

[61] Yu H (2013). Identification and polymorphism discovery of the cathelicidins, Lf-CATHs in ranid amphibian (*Limnonectes fragilis*). FEBS J..

[62] Ling G (2014). Cathelicidins from the Bullfrog *Rana catesbeiana* provides novel template for peptide antibiotic design. PLoS ONE.

[63] Hancock REW, Rozek A (2002). Role of membranes in the activities of antimicrobial cationic peptides. FEMS Microbiol. Lett..

[64] Rivas L, Luque-Ortega JR, Andreu D (2009). Amphibian antimicrobial peptides and Protozoa: Lessons from parasites. Biochim. Biophys. Acta Biomembranes.

[65] Luo XJ (2021). A novel anionic cathelicidin lacking direct antimicrobial activity but with potent anti-inflammatory and wound healing activities from the salamander *Tylototriton kweichowensis*. Biochimie.

[66] Ruppert KM, Kline RJ, Rahman MS (2019). Past, present, and future perspectives of environmental DNA (eDNA) metabarcoding: A systematic review in methods, monitoring, and applications of global eDNA. Glob. Ecol. Conserv..

[67] Gendron EM (2023). Nematode mitochondrial metagenomics: A new tool for biodiversity analysis. Mol. Ecol. Resour..

[68] Vekemans X (2021). Whole-genome sequencing and genome regions of special interest: Lessons from major histocompatibility complex, sex determination, and plant self-incompatibility. Mol. Ecol..

[69] Lau Q (2020). Expression changes of MHC and other immune genes in frog skin during ontogeny. Animals.

[70] Savage A, Zamudio K (2011). MHC genotypes associate with resistance to a frog-killing fungus. Proc. Natl. Acad. Sci. USA.

[71] Trujillo AL (2021). Spatiotemporal adaptive evolution of an MHC immune gene in a frog-fungus disease system. Heredity.

[72] Christmas MJ (2023). Evolutionary constraint and innovation across hundreds of placental mammals. Science.

[73] Kosch TA (2023). Comparative analysis of amphibian genomes: An emerging resource for basic and applied research. bioRxiv.

[74] Larson DJ (2014). Wood frog adaptations to overwintering in Alaska: New limits to freezing tolerance. J. Exp. Biol..

[75] van Beurden EK (1980). Energy metabolism of dormant Australian water-holding frogs (*Cyclorana platycephalus*). Copeia.

[76] Raaymakers C (2017). Antimicrobial peptides in frog poisons constitute a molecular toxin delivery system against predators. Nat. Commun..

[77] Aztekin C (2019). Identification of a regeneration-organizing cell in the Xenopus tail. Science.

[78] Barnett DW (2011). BamTools: A C++ API and toolkit for analyzing and managing BAM files. Bioinformatics.

[79] Sim SB (2022). HiFiAdapterFilt, a memory efficient read processing pipeline, prevents occurrence of adapter sequence in PacBio HiFi reads and their negative impacts on genome assembly. BMC Genomics.

[80] Cheng H (2021). Haplotype-resolved de novo assembly using phased assembly graphs with Hifiasm. Nat. Methods.

[81] Zhou C, McCarthy SA, Durbin R (2022). YaHS: Yet another Hi–C scaffolding tool. Bioinformatics.

[82] Durand NC (2016). Juicebox provides a visualization system for Hi–C contact maps with unlimited zoom. Cell Syst..

[83] Allio R (2020). MitoFinder: Efficient automated large-scale extraction of mitogenomic data in target enrichment phylogenomics. Mol. Ecol. Resour..

[84] Uliano-Silva M (2023). MitoHiFi: A python pipeline for mitochondrial genome assembly from PacBio high fidelity reads. BMC Bioinform..

[85] Afgan E (2018). The Galaxy platform for accessible, reproducible and collaborative biomedical analyses: 2018 update. Nucleic Acids Res..

[86] Flynn JM (2020). RepeatModeler2 for automated genomic discovery of transposable element families. Proc. Natl. Acad. Sci..

[87] Smit, A., Hubley, R., Green, P. *RepeatMasker Open-4.0*. 2013–2015.

[88] Kim D, Langmead B, Salzberg SL (2015). HISAT: A fast spliced aligner with low memory requirements. Nat. Methods.

[89] Bolger AM, Lohse M, Usadel B (2014). Trimmomatic: A flexible trimmer for Illumina sequence data. Bioinformatics.

[90] Pertea M (2015). StringTie enables improved reconstruction of a transcriptome from RNA-seq reads. Nat. Biotechnol..

[91] Kuo RI (2020). Illuminating the dark side of the human transcriptome with long read transcript sequencing. BMC Genomics.

[92] Kang Y-J (2017). CPC2: A fast and accurate coding potential calculator based on sequence intrinsic features. Nucleic Acids Res..

[93] Haas BJ (2013). De novo transcript sequence reconstruction from RNA-seq using the Trinity platform for reference generation and analysis. Nat. Protocols.

[94] Altschul SF (1990). Basic local alignment search tool. J. Mol. Biol..

[95] Altschul SF (1997). Gapped BLAST and PSI-BLAST: A new generation of protein database search programs. Nucleic Acids Res..

[96] Eddy SR (2011). Accelerated profile HMM searches. PLoS Comput. Biol..

[97] Whittington CM (2008). Defensins and the convergent evolution of platypus and reptile venom genes. Genome Res..

[98] Schutte BC (2002). Discovery of five conserved beta-defensin gene clusters using a computational search strategy. Proc. Natl. Acad. Sci. USA.

[99] Helbing CC (2019). Antimicrobial peptides from Rana [Lithobates] catesbeiana: Gene structure and bioinformatic identification of novel forms from tadpoles. Sci. Rep..

[100] Minh BQ (2020). IQ-TREE 2: New models and efficient methods for phylogenetic inference in the genomic era. Mol. Biol. Evol..

[101] Kalyaanamoorthy S (2017). ModelFinder: Fast model selection for accurate phylogenetic estimates. Nat. Methods.

[102] Jones DT, Taylor WR, Thornton JM (1992). The rapid generation of mutation data matrices from protein sequences. Comput. Appl. Biosci..

[103] Dayhoff M, Schwartz R, Orcutt B, Dayhoff M (1978). A model of evolutionary change in proteins. Atlas of Protein Sequence and Structure.

[104] Hoang DT (2017). UFBoot2: Improving the Ultrafast Bootstrap Approximation. Mol. Biol. Evol..

[105] Tamura K, Stecher G, Kumar S (2021). MEGA11: Molecular evolutionary genetics analysis version 11. Mol. Biol. Evol..

[106] Tomasinsig L, Zanetti M (2005). The cathelicidins—Structure, function and evolution. Curr. Protein Pept. Sci..

[107] Teufel F (2022). SignalP 6.0 predicts all five types of signal peptides using protein language models. Nat. Biotechnol..

[108] Shi Y (2020). Cathelicidin-DM is an antimicrobial peptide from *Duttaphrynus melanostictus* and has wound-healing therapeutic potential. ACS Omega.

[109] Mu L (2017). The first identified cathelicidin from tree frogs possesses anti-inflammatory and partial LPS neutralization activities. Amino Acids.

[110] Duckert P, Brunak S, Blom N (2004). Prediction of proprotein convertase cleavage sites. Protein Eng. Des. Sel..

[111] Gasteiger E (2003). ExPASy: The proteomics server for in-depth protein knowledge and analysis. Nucleic Acids Res..

[112] Huttner KM, Bevins CL (1999). Antimicrobial peptides as mediators of epithelial host defense. Pediatr. Res..

[113] Tang YQ, Selsted ME (1993). Characterization of the disulfide motif in BNBD-12, an antimicrobial beta-defensin peptide from bovine neutrophils. J. Biol. Chem..

[114] Semple F, Dorin JR (2012). β-Defensins: Multifunctional modulators of infection, inflammation and more?. J. Innate Immunity.

[115] Peel E (2017). Marsupial and monotreme cathelicidins display antimicrobial activity, including against methicillin-resistant *Staphylococcus aureus*. Microbiology.

